# Analgesia after tonsillectomy with controlled intravenous morphine — overdue or exaggerated?

**DOI:** 10.1016/j.bjorl.2021.08.002

**Published:** 2021-10-18

**Authors:** Magdalena Gostian, Johannes Loeser, Tanya Bentley, Philipp Wolber, David Schwarz, Matthias Balk, Antoniu-Oreste Gostian

**Affiliations:** a Uniklinik Koeln, Department of Anaesthesiology and Intensive Care Medicine, Koeln, Germany; b Malteser Waldkrankenhaus St. Marien, Department of Anaesthesiology and Intensive Care Medicine, Erlangen, Germany; c Uniklinik Koeln, Department of Palliative Medicine, Koeln, Germany; d Uniklinik Koeln, Department of Otolaryngology, Head & Neck Surgery, Koeln, Germany; e Universitaetsklinikum Erlangen, Department of Otolaryngology, Head & Neck Surgery, Erlangen, Germany

**Keywords:** Tonsillectomy, Postoperative pain, Patient reported outcome, Quality of life, Patient satisfaction

## Abstract

**Objective:**

To assess the value of a morphine Patient Controlled Intravenous Analgesia (PCIA) after Tonsillectomies (TE).

**Methods:**

30 adult patients were treated with oral analgesics (protocol group) and compared to 30 patients treated with a morphine PCIA for the first 3 Postoperative Days (PODs) after TE. Average and maximum pain severities (Numeric Rating Scale – NRS: 0–10) on PODs 1–3, analgesic score, quality of life, patient satisfaction and side effects were defined as outcome measures.

**Results:**

Average pain severities of the protocol and the PCIA group were of similar magnitude (NRS) (POD1: 4.48 vs. 4.71 [*p* = 0.68], POD2: 4.75 vs. 4.22 [*p* = 0.32] and POD3: 4.44 vs. 4.25 [*p* = 0.71]). Maximum pain intensities on POD1 (*p* = 0.92), POD2 (*p* = 0.51) and POD3 (*p* = 0.36) were also comparable between both groups. Patients with a PCIA consumed significantly more opioids (*p* = 0.001) without significant more side-effects.

**Conclusion:**

The PCIA did not provide a superior pain control compared to oral analgesics. In view of the considerable effort and the high opioid consumption, it cannot be recommended as a standardized application for pain control after TE.

## Highlights

• Postoperative pain intensities following tonsillectomy regularly reach high levels.
• So far there is no effective evidence-based treatment concept.
• Strong opioids for pain treatment have not been evaluated yet.
• Patient Controlled Intravenous Analgesia is effective in multiple disciplines.
• After tonsillectomy opioids are not a necessary and effective therapeutic option.

## Introduction

Postoperative pain intensities following Tonsillectomy (TE) regularly reach high levels affecting especially the first four Postoperative Days (PODs) and so far, there is no effective evidence-based treatment concept.^1^ Consequently, in view of the known detrimental physiological consequences of untreated acute postoperative pain there is a great need for improvement of this unsatisfactory issue. Following TE in adults, weak opioids allow for only limited pain relief but strong opioids have not been evaluated yet.^2^ Patient Controlled Intravenous Analgesia (PCIA) has proven to be effective and safe in multiple surgical disciplines alongside with a high patient satisfaction.^3^, ^4^, ^5^ Based on our clinical experience, we considered PCIA as the last treatment option in our department and thus restricted it to a few patients that sustained high postoperative pain levels despite oral analgesic treatment. Actually, we observed an effective pain reduction and a consistently high satisfaction in these patients.

Consequently, aiming at quality improvement of our postoperative pain treatment we hypothesized that a routine application of a PCIA with morphine combined with opioid-sparing peripherally acting analgesics may improve pain treatment after TE.

## Methods

This prospective study was approved by the Ethics Committee of the University of Cologne (application number: 16–190), registered in the German Clinical Trials Register (DRKS) (application number: DRKS00011092), was conducted according to the Declaration of Helsinki and followed the STROBE guidelines.

A total of 60 consecutive patients presenting for elective bilateral TE at the Department of Otorhinolaryngology-Head and Neck Surgery of the University of Cologne between September 1st, 2016 and November 1st, 2018 were included in the study. The following inclusion criteria were applied: bilateral total TE due to chronic recurrent tonsillitis following the criteria of the S2k guideline “Inflammatory diseases of the palatine tonsils/tonsillitis, therapy”,^6^ age ≥18 years, American Society of Anaesthesiologists (ASA) status I–III and a Body Mass Index (BMI) ≥18.5 kg/m^2^. Exclusion criteria were: single sided and partial TE, TE à chaud, oncologic TE, pre-existing opioid-dependent pain, central nervous system disease and Obstructive Sleep Apnoea (OSA). OSA was excluded on the basis of medical history. Patients were specifically asked about symptoms of OSA, previous sleep medical diagnosis and possible nocturnal CPAP therapy. Patients were also monitored in the recovery room for several hours after surgery. If saturation drops had occurred postoperatively, the patients would not have received PCIA with opioids.

**Figure 1:**
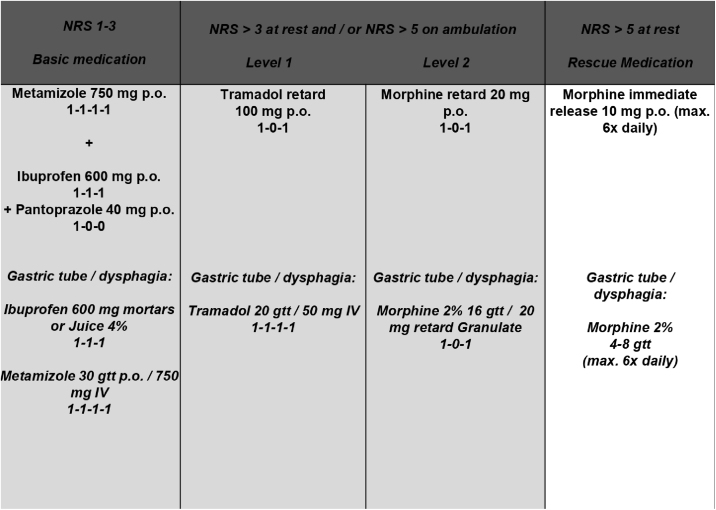
Applied oral pain concept. NRS, Numeric Rating Scale (0 = “no pain”–10 = “worst pain imaginable”); p.o., per os; IV, Intravenous; gtt, guttate.

All TEs were performed as “cold dissections” with blunt dissection along the tonsil’s capsule. General anaesthesia was induced with Propofol (2–3 mg × kg^−1^) and Fentanyl (2–3 µg × kg^−1^) and maintained with Sevoflurane (1.7–2.0 vol%) in an air-oxygen mixture combined with Fentanyl (1 µg × kg^−1^bolus as required). In case of infection, antibiotics were administered during POD 1–3 (sultamicillin 750 mg p.o., twice daily) (Table 1). Postoperatively, 30 consecutive patients (protocol group) were treated according to a standardized escalating oral pain concept representing the department’s current standard (Fig. 1). Subsequently, 30 consecutive patients (PCIA group) received a PCIA pump (CADD®-Solis v3.0 Ambulatory Infusion Pump; Smiths Medical Deutschland GmbH, Grasbrunn, Germany) postoperatively in the recovery room containing 100 mL of 1.0 mg/mL morphine delivering a bolus of 1 mg (5-minute lockout time; 30 mg maximum dose within 4 h; no loading/background infusion). It was removed on POD3. All patients routinely received the basic pain medication of the pain protocol and were visited twice daily by the medical staff. Because of allergies or chronic gastritis, ibuprofen and metamizole were refused to four patients of the protocol and one patient of the PCIA group, respectively. Opioid consumption was analysed by converting the intravenous opioid intake into Oral Morphine Equivalents (OME): “strength per unit × number of units × OME conversion factor (IV morphine: 3.0; tramadol p.o.: 0.2) = total OME units”.^7^ Primary endpoints were defined as average and maximum pain intensity on PODs 1–3. Secondary endpoints included pain tolerance, analgesic score, quality of life, patient satisfaction and treatment related side effects.

**Table 1:** Demographic and clinical characteristics of patients treated according to the escalating oral pain concept (protocol group) and with PCIA (PCIA group).

Parameter	Protocol group (n = 30)	PCIA group (n = 30)	Statistical comparison
Female gender (n/%)	19/63.3	18/60.0	χ^2^(1) = 0.07, *p* = 0.79
Age (years)	25.3 ± 5.1 (18–36)	24.9 ± 7.0 (18–45)	t_(58)_ = 0.27, *p* = 0.79
Height (cm)	172.8 ± 7.2	175.6 ± 11.4	t_(48,74)_ = −1.15, *p* = 0.26
Weight (kg)	76.8 ± 17.3	77.4 ± 14.3	t_(58)_ = −0.16, *p* = 0.86
ASA physical status (I/II/III) (n/%)	I: 20/66.7	I: 24/82.8	χ^2^(1) = 2.01, *p* = 0.16
	II: 10/33.3	II: 5/17.2	
Duration of inpatient stay	4.7 ± 1.6 (3–9)	4.4 ± 1.3 (3–11)	t_(58)_ = 0.71, *p* = 0.48
Cutting/Suture time (min)	37.7 ± 11.7 (12–57)	38.9 ± 13.6 (10–63)	t_(58)_ = −0.37, *p* = 0.72
Piritramide in the recovery room (mg)	2.3 ± 3.0 (0–8.5)	1.8 ± 4.3 (0–20)	t_(58)_ = 0.57, *p* = 0.57
Fentanyl intraoperatively (mg)	0.38 ± 0.10 (0.2–0.5)	0.41 ± 0.15 (0.25–1)	t_(58)_ = −0.92, *p* = 0.36
Antibiotics administered postoperatively (n/%)	3/10.0	7/23.3	χ^2^(1) = 1.92, *p* = 0.17

PCIA, Patient Controlled Intravenous Analgesia; ASA, American Society of Anesthesiologists.

Pain perception (Numeric Rating Scale – NRS: 0 = “no pain” – 10 = “worst pain imaginable”) and pain tolerance (NRS: 1 = “no pain”, 2 = “pain well tolerable”, 3 = “pain barely tolerable”, 4 = “pain unbearable”) were surveyed twice daily. Health-related quality of life was evaluated preoperatively and on POD3 using the “acute version” of the validated Short Form Survey (SF-36).^8^

Patient satisfaction and involvement in the analgesic treatment (NRS 0–10) were surveyed using the “Quality Improvement in Postoperative Pain Management” (QUIPS) questionnaire.^9^, ^10^ Incidence and severity (NRS 0–10) of treatment related side effects were evaluated daily.

Mixed-model ANOVAs with the between-factor “group” and the within-factor “time” computed the pain intensity related to treatment group. Paired or unpaired t-tests served as post-hoc tests with Bonferroni correction. Side effects were analysed using Chi-square, Fisher’s exact and unpaired *t*-tests, where applicable (IBM SPSS Statistics 23.0; IBM, New York, NY). Numerical values are presented as mean values ± standard deviation (SD). *p*-Values of <0.05 were considered statistically significant.

Sample size estimation was performed for the primary endpoint maximum and average pain perception and was based on Gostian et al. (2020).^14^ Here, two standardized analgesic treatment protocols were compared regarding postoperative pain perception after tonsillectomy. A medium sized effect was found for maximum pain (f = 0.251) and pain on ambulation (f = 0.223). Transferred to our targeted effect POD × group, a sample size of at least 28 patients per group would be required to detect differences between both treatment groups.

## Results

The study included 60 out of 64 eligible candidates. Two patients withdrew their initial consent, one patient discharged himself earlier and for one patient clinical data was incomplete. Demographic and clinical characteristics revealed no statistically significant differences between both patient groups (Table 1).

**Figure 2:**
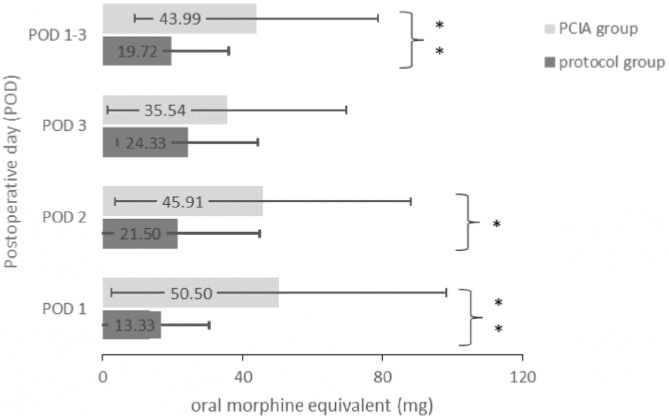
Postoperative opioid consumption on POD 1–3. Values represent the mean amount of administered opioid medication given in mg Oral Morphine Equivalents on each POD and averaged over POD 1–3. POD, Postoperative Day; PCIA, Patient Controlled Intravenous Analgesia; OME, Oral Morphine Equivalent.

**Figure 3:**
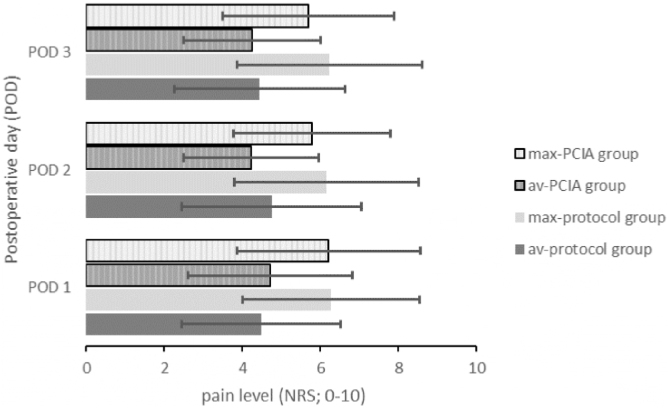
Average and maximum pain intensities of both study groups reported on each POD 1–3. Data presented as average value of all patients included. Av, Average pain level; max, Maximum pain level; NRS, Numeric Rating Scale; PCIA, Patient Controlled Intravenous Analgesia; POD, Postoperative Day.

Opioids were applied to 22 patients of the protocol group (73.3%) and to all 30 patients of the PCIA group (100%) (*p* = 0.04). As a consequence, the average opioid consumption over all three PODs was significantly higher in the PCIA group (43.99 ± 34.80 mg) compared to the protocol group (19.72 ± 16.36 mg; t_(58)_ = −3.46, *p* = 0.001). Fig. 2 displays the daily and average opioid consumption. The reported average pain intensities were of similar magnitude (F(1.69,97.83) = 1.29, *p* = 0.28 with POD1: t(58) = −0.420, *p* = 0.676, POD2: t(58) = 1.011, *p* = 0.316 and POD3: t(58) = 0.374, *p* = 0.710). Similarly, maximum pain intensities were independent of the application of the PCIA (F(2,116) = 0.45, *p* = 0.64 with POD1: t(58) = 0.098, *p* = 0.922, POD2: t(58) = 0.663, *p* = 0.510 and POD3: t(58) = 0.919, *p* = 0.362) (Fig. 3). Patients with a PCIA reported significantly favourable pain tolerance on POD 3 (2.38 vs. 2.71; t_(51,5)_ = 2.216, *p* = 0.031) only.

Patients receiving a PCIA complained more often of obstipation (χ^2^(1) = 5.55, *p* = 0.02) at a similarly low level compared to patients of the protocol group (t_(32)_ = 1.39, *p* = 0.17). Furthermore, incidences and severities of the surveyed treatment related side effects were comparable in both patient groups (Table 2).

**Table 2:** Incidence and degree of treatment-related side effects of all patients. The degree of each side effect is given based on the patient reported value according to an NRS ranging from 0 (minimal value) to 10 (maximal value).

Side effect	Protocol group (n = 30)	PCIA group (n = 30)	Statistical comparison
Persistent nausea (n/%)	20/66.7	19/63.3	*p* = 0.79
Degree of persistent nausea	2.30 ± 1.39	3.15 ± 2.03	t_(37)_ = 1.54, *p* = 0.13
Vomiting (n/%)	7/23.3	10/33.3	*p* = 0.39
Degree of vomitting	2.26 ± 1.42	1.97 ± 1.67	t_(15)_ = 0.38, *p* = 0.71
Obstipation (n/%)	13/43.3	22/73.3	*p* = 0.02
Degree of obstipation	1.55 ± 1.07	2.44 ± 2.15	t_(32)_ = 1.39, *p* = 0.17
Fatigue (n/%)	28/93.3	28/93.3	*p* > 0.999
Degree of fatigue	4.25 ± 1.98	4. 23 ± 2.23	t_(44)_ = 0.032, *p* = 0.98
Concentration disorders (n/%)	14/46.7	21/70.0	*p* = 0.07
Degree of concentration disorders	2.76 ± 1.85	2.54 ± 1.76	t_(33)_ = 0.36, *p* = 0.72
Sleep disturbance (n/%)	29/96.7	26/86.7	*p* = 0.16
Degree of sleep disturbance	2.53 ± 1.77	2.89 ± 1.82	t_(53)_ = −0.76, *p* = 0.45
Vertigo (n/%)	18/60.0	17/56.7	*p* = 0.79
Degree of vertigo	2.11 ± 1.53	2.70 ± 2.12	t_(33)_ = −0.95, *p* = 0.35

**Figure 4:**
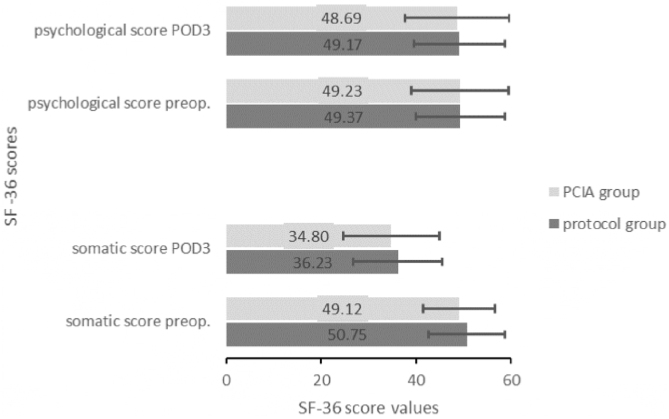
Somatic and psychological scores of both study groups preoperatively and on POD3. PCIA, Patient Controlled Intravenous Analgesia; POD, Postoperative Day.

Similar somatic (preoperative: protocol group = 50.75 ± 8.04 vs. PCIA group = 49.12 ± 7.6; t_(57)_ = 0.802, *p* = 0.426; POD3: 36.23 ± 9.41 vs. 34.8 ± 10.21; t_(58)_ = 0.671, *p* = 0.505) and psychological scores (preoperative: protocol group = 49.37 ± 9.42 vs. PCIA group = 49.23 ± 10.27; t_(57)_ = 0.135, *p* = 0.893; POD3: 49.17 ± 9.5 vs. 48.69 ± 11.09; t_(58)_ = 0.135, *p* = 0.893) indicated that health-related quality of life was irrespective of the applied treatment (Fig. 4).

All surveyed patients felt equally involved in their pain management (protocol group: 8.43 ± 1.68 vs. PCIA group: 7.66 ± 1.99; t_(57)_ = −1.63, *p* = 0.11) and were comparably satisfied with their analgesic treatment (protocol group: 7.90 ± 1.79 vs. PCIA group: 7.38 ± 2.65; t_(51,36)_ = −0.90, *p* = 0.38).

## Discussion

This study shows that mean values of the reported average and maximum pain intensities did not differ significantly between both analgesic treatments. This is all the more surprising since patients of the PCIA group consumed twice as much opioids compared to the protocol group as expected and known from various PCIA applications, where in contrast to our study, pain scores were usually lower with higher analgesic use.^5^

Apparently, average and maximum pain levels were rather high throughout all days. Numerous studies have already demonstrated that pain intensities after TE regularly reach very high intensities.^1^ Accordingly, the German S3-guidelines on treatment of acute perioperative and posttraumatic pain explicitly recommend the use of strong opioids in case of expected severe postoperative pain and the application of PCIA systems within a balanced analgesic concept.^3^ In view of the reported benefits of PCIA and the expected problems with swallowing, the use of morphine-based PCIA after TE was considered to be a viable option.

Still, the results in pain relief after TE remained unsatisfactory. This may be attributed to the lack of knowledge on the exact cause of this severe pain. It is assumed to be either of inflammatory and/or neuropathic origin and there might be a pain component that is non-opioid sensitive. In this regard, further studies in order to identify the underlying pathophysiological mechanism of pain after TE and to implement potential new therapeutic targets are desperately needed in order to improve the unsatisfactory issue of pain treatment after TE.

Our work is in line with the study of Kelly et al., where it was demonstrated that using morphine after TE in children did not lead to a superior pain control but to an increase in serious and dangerous side effects in children.^11^ In our study collective the overall occurrence of complications and side effects were comparably low as expected from previous studies in adults.^4^ Still, the missing benefit of using opioids for pain control after TE was identical.

Receiving a PCIA pump for pain relief was assessed positively by the patients but did not lead to a higher patient satisfaction compared to oral analgesics. It is known that there seems to be only a weak correlation between analgesia and patient satisfaction and patients rate their satisfaction high despite of having substantial pain.

Limitations of our study are based on the missing randomization. We still believe that both groups were highly comparable, since both treatment groups were similar in regard to demographic and clinical factors out of which female gender, preoperative chronic pain and younger age contribute to increased postoperative pain sensation.^12^ Furthermore, bias caused by a placebo effect due to the PCIA cannot be excluded with any certainty. In this regard, all patients were visited twice daily by the acute pain service in order to minimize bias due to attention, documentation, patient involvement and sufficient information, which are all known factors contributing to a better pain management and higher patient satisfaction.^13^

## Conclusion

In conclusion, this pioneer survey on the efficacy of morphine PCIA to treat postoperative pain after TE in adults shows that opioids are not a necessary and effective therapeutic option. Further studies in order to identify the underlying pathophysiological mechanism of pain after TE and possible new therapeutic targets are desperately needed in order to improve the unsatisfactory issue of pain treatment after TE.

## Conflicts of interest

The authors declare no conflicts of interest.
